# Cyber-physical oil spill monitoring and detection for offshore petroleum risk management service

**DOI:** 10.1038/s41598-023-30311-w

**Published:** 2023-03-20

**Authors:** Yuewei Wang, Xiaodao Chen, Lizhe Wang

**Affiliations:** School of Computer Science, China University of Geosciences, Wuhan, 430078 China

**Keywords:** Natural hazards, Ocean sciences

## Abstract

Petroleum industry has started to embrace the advanced petroleum cyber-physical system (CPS) technologies. Offshore petroleum CPS is particularly hard to build, mainly due to the difficulty in detecting and preventing offshore oil leaking. During the oil exploration and transportation process, the remote multi-sensing technology is typically employed for emerging service. It can be utilized for leak detection by enabling the underwater modeling of an offshore petroleum CPS. However, such a technology suffers from insufficient remote sensing resources and expensive computational overhead. In this work, a cross-entropy based leak detection technique is proposed to detect the oil leak, which facilitates the understanding of the oil leak induced marine pollution. Furthermore, a hierarchical parallel approach is proposed on the super computer Tianhe-2 to improve the efficiency of the proposed leak detection technique. Experimental results on Penglai oil spill events demonstrate that the proposed method can effectively identify the sources of oil spilling with accuracy up to $100\%$.

## Introduction

Advanced petroleum cyber-physical systems (CPS) technologies have been adopted in various activities in the petroleum industry where petroleum CPS optimization tools can facilitate the processes of petroleum exploration, production and management^1,2^. For example, there are studies on interwell connectivity of petroleum reservoir^3^ and cyberattack analysis on the Oil and Gas ($O \& G$) field^4^. For these petroleum CPSs, real-time production data, together with static geological data, are employed to improve the petroleum system. In this work, we focus on the offshore petroleum CPS to detect and prevent offshore oil leaking.

The oil leaking events occur during oil exploration and transportation in the offshore petroleum industry. Oil leak can induce significant pollution in the ocean, which leads to various economical and environmental issues^5,6^. Thus, a petroleum CPS which can allocate the leak point effectively for oil leak detection is highly demanded^7^. However, locating the leaking point needs significant work due to the reason that the underwater oil spilling model is very complicated which can be impacted by a number of factors such as chemical transformations and biological consumption of the oil. This simulation of tracking oil at both vertical and horizontal is with features of nonlinear, time-varying and multi-variables^8^, such that, the model itself is significant complex. For this kind of model, it is difficult to directly locate the leaking point by utilizing the model inversely. However, in industry, an accurate leaking point estimation serves as a very important role for the emergency response of petroleum CPS^9,10^. Collaborate the plain offshore oil leaking model needs to be improved by other techniques for the target problem.

**Figure 1:**
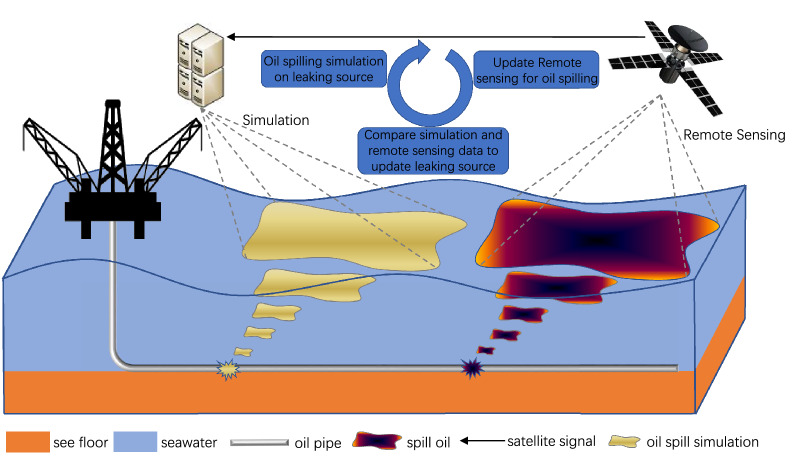
Illustration on remote sensing helped offshore oil spill monitoring and detection in offshore petroleum CPS based emerging service.

During oil exploration and transportation, the remote multi-sensing technology is typically used for leak detection^11^, enabling the underwater modeling of an offshore petroleum CPS based emerging service as shown in Fig. 1. The oil leaking accident is detected which can be analyzed using the remote sensing data. Synthetic Aperture Radar (SAR) is an effective and widely utilized remote sensing technique for oil spill detection^12,13^. During the detection procedure, an assumed oil leak source can be placed in a numerical simulation; and can be evaluated by comparing the simulation results with remote sensing data. After that, the assumed oil leak source can be updated which targets to a better similarity to the remote sensing data. This procedure proceeds iteratively. Since satellites have their fixed and long revisit time, monitoring and detecting oil spill cannot rely only on the remote sensing data. The oil spilling simulation is normally combined with remote sensing technique. There are existing work on numerical oil models combining remote sensing data to provide continuous monitoring and detection of oil spill diffusion. In^14^, the combination of satellite data of oil slicks surface with Lagrangian trajectory models have been proposed for trajectory forecast on oil leaking. In^15^, the MEDSLIK oil spill model is proposed to forecast the oil spreading situation by analyzing the remote sensing image^16^. It was later improved to the MEDSLIK-II Lagrangian marine model which can be utilized to simulate the spread and transformation processes of leaking oil by using both SAR (synthetic aperture radar) data and optical image data^17^. In^18^, an Estuarine Coastal Ocean Model (ECOM-3d) was established by combining a remote sensor system that detects oil spills with a numerical simulation based on a dynamic data-driven application system. These existing techniques suffer from insufficient remote sensing resources and expensive computational cost, when working with realistic scenarios.

In this work, a new technique is proposed for offshore oil spill monitoring and detecting based on the numerical simulation and the remote sensing data. Leveraging the cross entropy technique, the proposed technique determines the offshore oil linking, which can also facilitate the understanding of the oil leak induced marine pollution and enabling the better solutions. To significantly improve the efficiency of the proposed method, a hierarchical parallel approach is proposed to speed up the proposed method on super computer. Contributions of this paper are summarized as follows.

- A cross entropy optimization technique is developed for the offshore oil spill detection, to understand of the oil leak induced marine pollution.
- The unstructured grid Finite Volume Community Ocean Model (FVCOM) based on unstructured triangle grid is utilized to simulate oil leak process, to fit complex terrain and improve the simulation accuracy.
- A hierarchical parallel approach is proposed to deploy the oil leak source detection method on super computer Tianhe-2. In this way, the in time responses of offshore petroleum detection CPS system can be enhanced for the further disaster prevention.
- The proposed technique is applied to analyze the real oil spill event in Penglai, China. Our case study demonstrates that the proposed method can effectively identify the sources of oil spilling with accuracy up to $100\%$.

Compared to the conference version of this paper^19^ which proposed a petroleum spill source detection method on a single computation node, this paper significantly extends it to develop a hierarchical parallel computing technique for oil spill detection. To leverage the technique of parallel computing, the numerical model of oil spilling is also changed from the ECOM-3d to the FVCOM. FVCOM, based on unstructured triangle grid, can simulate the complex coastline and massive diversity island in offshore region with accuracy. In addition, the improved method has been deployed to the super computer Tianhe-2 to meet the service efficiency for the offshore petroleum risk management. Sufficient computing on resource Tianhe-2 supports the numerical simulation and spill source detection with massive parallel execution. The proposed hierarchical parallel computing technique in this paper significantly increases the efficiency of the emerging service to make in time responses for oil leak accident.

## Preliminaries

FVCOM is an unstructured grid, primitive governing equations based coastal ocean and estuarine numeric simulation model. It was developed by Chen et al. in 1999^20^. FVCOM originally targeted at solving the 3-D current simulation; and current version of FVCOM imbibes advantages of traditional ocean circulation models, the finite-difference method and the finite-element method. By the help of its discretized method for governing integral equation, the accuracy and efficiency of the simulation can be achieved. With increasing number of researchers participated in FVCOM development, FVCOM has been extended with components including sea ice module, surface wave module, generalized biological module, 3-D Lagrangian particle tracking module, et al. Leveraging by the high performance computing technique, FVCOM can be parallel executed on computing clusters. Due to aforementioned metrics of FVCOM, it has became a widely used ocean numerical simulation model. In this paper, FVCOM is utilized to simulate the oil particle movement in an oil leak accident^21^.

**Figure 2:**
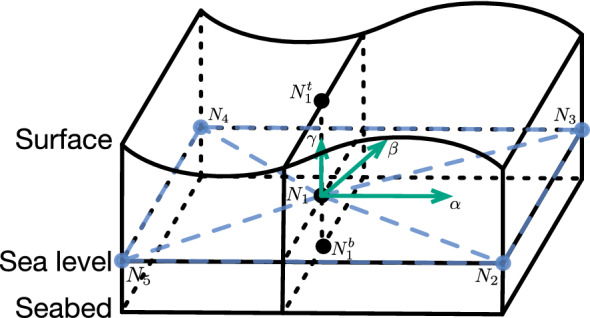
Illustration of a grid instance. $N_i$ represents the node *i*, $N_i^t$ and $N_i^b$ represent the corresponding node *i* on sea surface and seabed.

Numerical equations in FVCOM include conservation equations, momentum equations, temperature, salinity, and density equations. Conservation equations are built to simulate the movement of water in mathematics. Unstructured triangular grids are the basic units to build conservation equations. Momentum, continuity, temperature, density, salinity conservation equations are formulated to describe the dynamic relationship between grids. As shown in Fig. 2, four triangle grid instances are created at sea level to discretize the marine region for further dynamic analysis. The unstructured, non-overlapped grids can flexibly deal with complex terrain^22^. For a node $N_i$ on sea level, the distance of the node $N_i$ to its projection node $N_i^t$ on ocean surface is $\delta$; and the distance of the node $N_i$ to its projection node $N_i^b$ on seabed is *h*. There are scalar factors are associated to each node which can be utilized to illustrate the state of the ocean system. These scalar factors are: the seabed depth *h* (sea-level standard), the surface height $\delta$ (sea-level standard), the *z* velocity component $\gamma$, the salinity *S*, temperature *T*, density $\rho$, vertical eddy coefficient and thermal vertical eddy diffusion coefficient $\kappa$ and $\kappa _T$. Besides, *x*, *y* and *z* velocity components $\textbf{v}\left( \alpha , \beta , \gamma \right)$ are placed at centroids of each triangular gird. For each triangular grid, the continuity equation is:

1 $$\begin{aligned} \frac{\partial \alpha }{\partial x} + \frac{\partial \beta }{\partial y} + \frac{\partial \gamma }{\partial z} = 0. \end{aligned}$$

The momentum equations are formulated as:

2 $$\begin{aligned} \begin{aligned} \frac{\partial \alpha }{\partial t} + \textbf{v}\cdot \nabla \alpha - C\beta&= - \frac{1}{\rho }\frac{\partial P}{\partial x} - \frac{1}{\rho }\frac{\partial q}{\partial x} + \frac{\partial }{\partial z}\left( \kappa \frac{\partial \alpha }{\partial z}\right) + F_{\alpha } \\ \frac{\partial \beta }{\partial t} + \textbf{v}\cdot \nabla \beta - C\alpha&= - \frac{1}{\rho }\frac{\partial P}{\partial y} - \frac{1}{\rho }\frac{\partial q}{\partial y} + \frac{\partial }{\partial z}\left( \kappa \frac{\partial \beta }{\partial z}\right) + F_{\beta } \\ \frac{\partial \gamma }{\partial t} + \textbf{v}\cdot \nabla \gamma&= - \frac{1}{\rho }\frac{\partial P}{\partial z} + \frac{\partial }{\partial z}\left( \kappa \frac{\partial \gamma }{\partial z}\right) + F_{\gamma }. \end{aligned} \end{aligned}$$

where $F_{\alpha }, F_{\beta }$ and $F_{\gamma }$ represent the horizontal and vertical momentums in different direction. *P* represents the pressure which includes air pressure at ocean surface and hydrostatic pressure. *q* represents non-hydrostatic pressure. *C* represents the Coriolis parameter. The temperature, salinity, and density equations are as follows^21^.

3 $$\begin{aligned} \begin{aligned} \frac{\partial T}{\partial t} + \textbf{v}\cdot \nabla T =&\frac{\partial }{\partial z}\left( \kappa _T\frac{\partial T}{\partial z}\right) + F_T \\ \frac{\partial S}{\partial t} + \textbf{v}\cdot \nabla S =&\frac{\partial }{\partial z}\left( \kappa _T\frac{\partial S}{\partial z}\right) + F_S \\ \rho =&\rho (T, S, P+q) \end{aligned} \end{aligned}$$

where $F_T$ and $F_S$ represent the thermal and salt diffusion parameters. The numerical procedure is described by the above partial differential equations, which are solved by the semi-implicit solver.

Based on the velocity field created by conservation equations, the Lagrangian particle tracking module continuously releases petroleum particles. The dynamic movements of each particles can be described by ordinary differential equations^21^.

4 $$\begin{aligned} L'_n(t) = \textbf{v}\left( L_n, t\right) \end{aligned}$$

where $L_n(t)$ is the particle *n* location at a time *t*. $L'_n$ is the speed of the particle *n*. $\textbf{v}$ is the velocity field calculated by the model. The temporal movement is determined by the location of the particle and the time. To tackle the problem, the formula can be transformed into discrete integral^21^:

5 $$\begin{aligned} L_n(t') = L_n(t) + \int _{t}^{t'}\textbf{v}(L_n(t), \varphi ){\textbf{d}}\varphi . \end{aligned}$$

The equation discretizes the time interval and cumulative velocity to obtain the particle locations. The 4-stage explicit Runge-Kutta method is utilized to solve the integral problem. For more details regarding the Lagrangian particle tracking module of FVCOM, please refer to literature^21^.

## Methods

Based on oil leak numeric simulation model illustrated in the last section, in this section, the details of the proposed method are introduced as follows. The fundamental theoretical foundation for the cross entropy optimization method, the workflow of the proposed method while applying in solving the oil leak source detection problem, and the parallel method are introduced as follow.

### Theoretical foundation

Cross entropy (CE) optimization method is essentially an important sampling based Monte Carlo approach for optimization. The CE method is a relatively efficient technique for tackling complex optimization problems^23^. It treats an optimization problem as a rare event searching problem which can be achieved through iteratively tuning the Probability Density Function (PDF) of solutions. In this paper, the probability of the physical location of possible oil leak source is treated as a rare event. The CE method are utilized to efficiently locate the leaking point positions. The principle of the CE method is illustrated in this section. Cross-Entropy (CE) method is an important sampling based Monte Carlo approach for optimization.

For optimization problems, tentative solutions can be presented as $X^v_1, X^v_2,\ldots , X^v_n$. Those solutions can be generated in the decision domain $\xi$ according to the PDF $f(X^v,v)$ where *v* is the initial PDF characterizing parameter. Note that, since the solution PDF is unknown at the beginning, the $f(X^v,v)$ can be initialized by experience and it is to be tuned to obtained the solution PDF in this technique. After generating solutions $X^v_1,X^v_2,\ldots ,X^v_n$, they are applied into the problem to obtain corresponding results. Assume that the target optimization problem is to maximize the result value and can be presented as:

6 $$\begin{aligned} \begin{array}{l} \gamma ^* = \max \limits _{X^v \in \xi }S\left( X^v\right) , \end{array} \end{aligned}$$

where *S*(*x*) is a real-valued function to calculate the result and $\gamma ^*$ denotes the maximum result among all possibly solutions. If the parameter *v* in the PDF $f(X^v,v)$ is set to *u*, a new set of random samples $X^u = (X^u_1, X^u_2,\ldots , X^u_n)$ can be generated. The later generated solutions can be evaluated as:

7 $$\begin{aligned} \begin{array}{l} \Delta q = S(X^u)-\gamma , \end{array} \end{aligned}$$

where $\Delta q$ is the quality of a solution vector $X^u$, $\gamma$ is a real number. The probability of which $S(X^u)$ is greater than $\gamma$ can be defined as

8 $$\begin{aligned} \begin{array}{l} {l} = P_u(\Delta q\ge 0)=E_u(I_{\{\Delta q\ge 0\}}), \end{array} \end{aligned}$$

where $E_u$ is the expectation and I(*) is the indicator function, which can map the solution vectors to the targeted problem $S(X^u)$ and evaluate the solution qualities. For instance, $I_{\{\Delta q\ge 0\}}$ equals to 1 if and only if $\Delta q\ge 0$.

The target of the CE method is to minimize the upper bound which is real number $\gamma$ on the problem function $S(X^u)$. In that case, under high probability, $\Delta q$ is less than 0 and $\gamma$ is the maximum value $\gamma ^*$ of the problem. In order to compute *l* for a given $\gamma$, crude Monto Carlo algorithm is a practical method. Numerous samples are generated from solution domain $\xi$ to estimate *l* as

9 $$\begin{aligned} \begin{array}{l} {{\widehat{l}}} = \frac{1}{N}\sum _{i=1}^{N}I_{\{\Delta q_i\ge 0\}}, \end{array} \end{aligned}$$

where $\Delta q_i$ is the quality of a solution vector $X_i$. Estimating *l* accurately requires a large quantity of samples which would bring in the high computational overhead, especially when $\Delta q_i\ge 0$ is a rare event. To tackle this problem, the CE method utilizes the important sample approach to avoid significant number solutions. The CE method defines a different PDF *h*(*X*, *w*) on solution domain $\xi$ for important samples. Calculate the ${{\widehat{l}}}$ combined with *h*(*X*, *w*) to estimate *l* as:

10 $$\begin{aligned} \begin{array}{l} {{\widehat{l}}} = \frac{1}{N}\sum _{i=1}^{N}I_{\{\Delta q\ge 0\}}\frac{f(X^u_i,u)}{h(X^u_i,w)}. \end{array} \end{aligned}$$

The PDF *h*(*X*, *w*) is defined as:

11 $$\begin{aligned} \begin{array}{l} h(X,w)=\frac{I_{\{\Delta q\ge 0\}}f(X,u)}{l}. \end{array} \end{aligned}$$

The Eq. (10) can be converted into:

12 $$\begin{aligned} \begin{array}{l} {{\widehat{l}}} = \frac{1}{N}\sum _{i=1}^{N}I_{\{\Delta q\ge 0\}}\frac{f(X^u_i,u)l}{I_{\{\Delta q\ge 0\}}f(X^u_i,u)}=l. \end{array} \end{aligned}$$

The PDF *h*(*X*, *w*) is still difficult to calculate while the $\widehat{l}$ is unknown. The CE method provide a distance computation method for two PDF h(X,w) and f(X,v) utilizing Kullback-Leibler distance. The definition of the Kullback-leibler distance is:

13 $$\begin{aligned} \begin{array}{l} D(h,f)=E_{h}ln\frac{h(X,w)}{f(X,v)}\\ \\ =\int h(X,w)lnh(X,w)dx-\int h(X,w)lnf(X,v)dx. \end{array} \end{aligned}$$

To obtain the minimize Kullback-leibler distance between *h*(*X*, *w*) and *f*(*X*, *v*), the *w* requires to:

14 $$\begin{aligned} \begin{array}{l} \max \limits _v\int h(X^v,w)lnf(X^v,v)dx. \end{array} \end{aligned}$$

Combine the Eq. (11) and the Eq. (14). There is:

15 $$\begin{aligned} \begin{array}{l} \max \limits _v \int \frac{I_{\{\Delta q\ge 0\}}f(X^v,v)}{l}lnf(X^v,v)dx \end{array} \end{aligned}$$

which can be simplified as:

16 $$\begin{aligned} \begin{array}{l} \max \limits _v D=\max \limits _v E_uI_{\{\Delta q\ge 0\}}lnf(X^u,v). \end{array} \end{aligned}$$

Due to the important sample is utilized in Eq. (16), the Eq. (16) can be written as:

17 $$\begin{aligned} \begin{array}{l} \max \limits _v D=\max \limits _v E_pI_{\{\Delta q\ge 0\}}\frac{f(X^u,u)}{f(X^u,p)}lnf(X^u,v). \end{array} \end{aligned}$$

According to the Eq. (17), any reference parameter *p* can make the equality holds. In order to obtain the value of the variable *v*, the Eq. (17) can be changed into:

18 $$\begin{aligned} \begin{array}{l} v=arg\max \limits _vE_pI_{\{\Delta q\ge 0\}}\frac{f(X^u,u)}{f(X^u,p)}lnf(X^u,v). \end{array} \end{aligned}$$

The estimation value of *v* can be calculated as:

19 $$\begin{aligned} \begin{array}{l} v=arg\max \limits _v\frac{1}{N}E_p\sum _{i=1}^{N}I_{\{\Delta q\ge 0\}}\frac{f(X^u_i,u)}{f(X^u_i,p)}lnf(X^u_i,v), \end{array} \end{aligned}$$

where the samples $X_1,X_2,\ldots ,X_n$ are generated by the PDF *f*(*X*, *p*). *p* can be calculated by taking the derivative as:

20 $$\begin{aligned} \begin{array}{l} \frac{1}{N}E_p\sum _{i=1}^{N}I_{\{\Delta q\ge 0\}}\frac{f(X^u_i,u)}{f(X^u_i,p)}\bigtriangledown lnf(X^u_i,v)=0. \end{array} \end{aligned}$$

**Figure 3:**
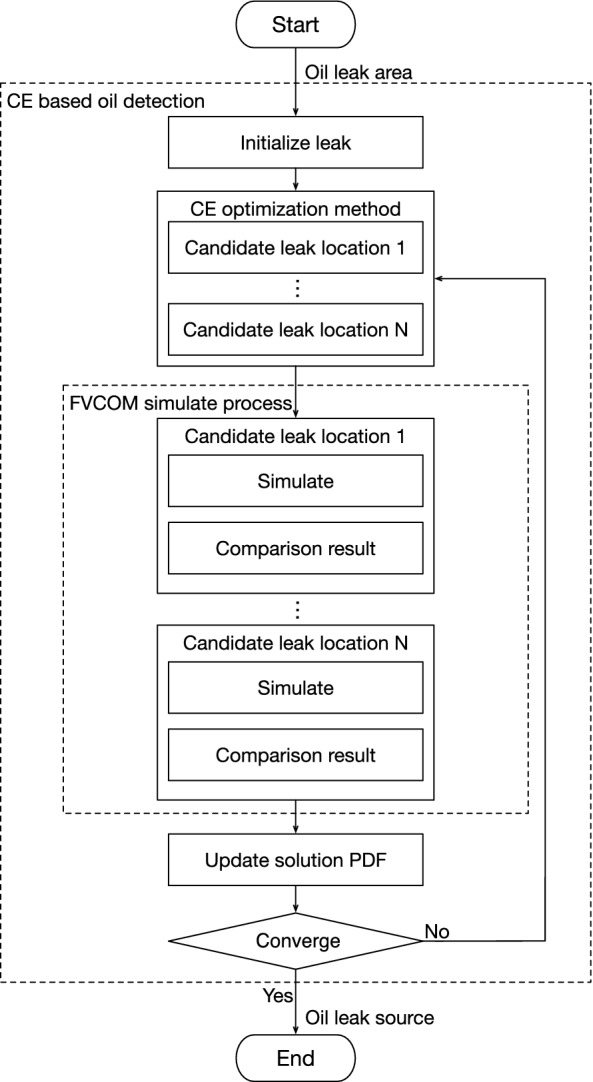
Illustration of the COSSD method workflow.

### CE based Offshore Oil Spill Detection Method

To obtain the accurate coordinates of the oil spill position, the CE based Oil Spill Source Detection (COSSD) method for offshore petroleum field is proposed in this work. It proceeds iteratively and in each iteration, a number of candidate leak sources are generated according to the solution PDF. For each candidate leak source, an oil spilling scenario is simulated and the polluted area on the surface of seawater can be obtained by the simulation. The simulation results are used in the candidate leak source evaluation. The evaluation is based on the mismatch value between the polluted area on the surface by the simulation and the area through analyzing the remote sensing satellite. Candidate leak sources with small mismatch values are considered as good quality samples and can be used to update the solution PDF in the next iteration.

The workflow of the COSSD method is shown in Fig. 3. Firstly, initialize the candidate oil leak sources PDF in the offshore region. A large number of candidate oil leak sources are generated by the initial PDF. Each candidate oil leak source is fed into the FVCOM model to simulate oil leak. Oil leak simulation results are compared to the remote sensing data to determine the qualities of the candidate oil leak sources. According to the simulation qualities, elite simulations can be selected to update the PDF of candidate oil leak sources. The simulation process is iteratively executed until the stop criteria being achieved. At the end of the proposed algorithm, the oil leak source location is obtained.
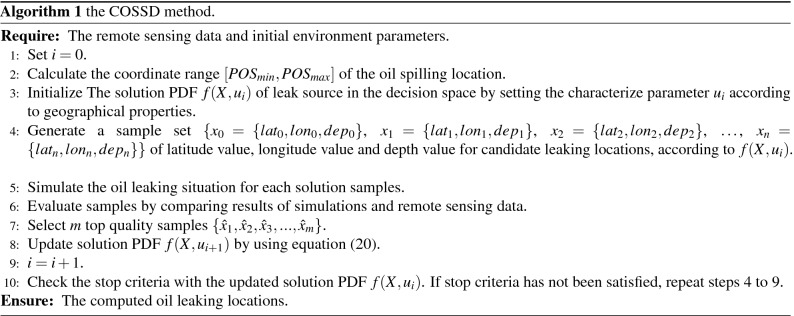


The details of the proposed COSSD method is illustrated in Algorithm 1. The input of the proposed algorithm is the remote sensing data and geological related parameters. At the beginning of the algorithm, the searching range for oil spill source and the location PDF $f(X,u_i)$ for the leak source are initialized in *step* 2 and *step* 3. Note that the search zone for the leak source and the solution PDF are initialized according to the geographical properties. In *step* 4, samples of candidate leak sources are generated according $f(X,u_i)$. These candidate leak sources are simulated in *step* 5. In this step, samples of leak sources can be simulated in parallel, since the scenarios are independent from each other. The simulation results of leak sources are evaluated in *step* 6 by comparing the simulation results and the remote sensing data. The leak sources of which simulation results that are close to remote sensing data can be treated as good quality samples. In *step* 7, top *m* samples which denoted by $\{{\hat{x}}_1, {\hat{x}}_2, {\hat{x}}_3,\ldots , {\hat{x}}_m\}$ are selected, which are used to update the solution PDF $f(X,u_{i+1})$ by equation (20) in *step* 8. In *step* 10, the stop criteria, which is the quality of the top sample, is checked to decide if the next iteration is necessary. If the simulation results by the top sample are close to the remote sensing results, the leak source can be determined. Otherwise, the next iteration (from *step* 4 to *step* 9) needs to be performed.

The COSSD method solves a rare event searching problem by important sampling. The sampling evaluation in every iteration is based on the simulation result. As the principle of the FVCOM mentioned above, the dynamic analysis consists of PDEs and ODEs, which bring in a large computational expense and are highly time-consuming^24,25^. Thus, to guarantee the efficiency of the proposed COSSD method, a parallel technique is necessary.

### A hierarchical parallel approach for the COSSD method executing on super computer

To improve the performance of the proposed COSSD method, super computers are employed to improve the computation efficiency.

The super computer can be divided into the login nodes and compute nodes. Users can access the login node and compute node by the network connection protocols. Login nodes have authority to apply for the specific number of cores to utilize. After login and basic operation environment initialization, task batches are submitted to compute nodes. By the help of multi-processors each compute node, computing tasks can be massively parallel executed on the super computer.

**Figure 4:**
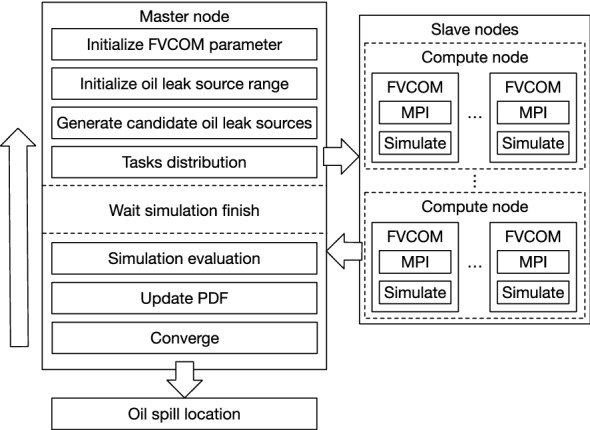
Illustration of the COSSD method parallel workflow.

**Figure 5:**
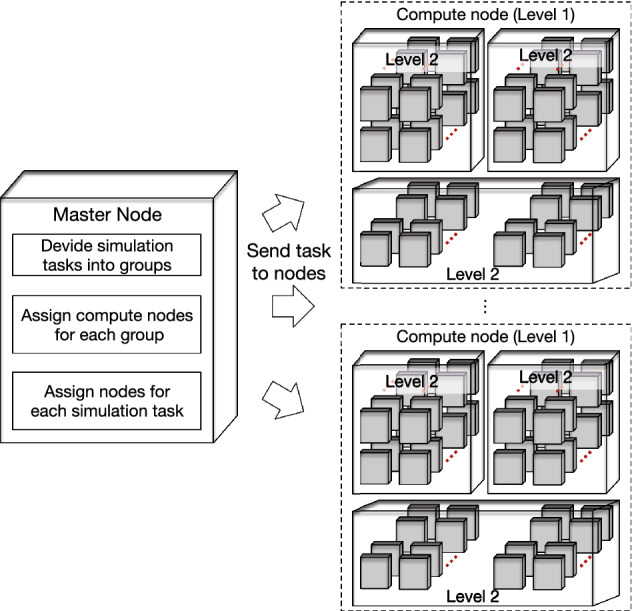
Illustration of multiple simulation tasks scheduling on the super computer.

Targeting at efficiency of parallel execution, a parallel task scheduling approach is proposed in this paper to fully embrace the advanced hardware architecture. The parallel task scheduling approach is shown in Fig. 4. Firstly, on the master node, for the FVCOM simulation requests, ocean factors which include the current field, wind field, salinity, temperature are initialized. After that, potential leak source regions are also initialized on the master node based on primary solution space. The potential leak source regions can be initialized at a coarse level. In the meantime, the corresponding oil leak source distribution PDF is initialized and a number of candidate leak sources are generated in the potential region. To significantly speed up computing part, the evaluation of the candidate oil leak source locations, which is most time-consuming in the proposed COSSD method, is distributed to the multiple slave nodes. Each slave node is assigned to process multiple FVCOM simulations. Note that, as shown in Fig. 5, these multiple FVCOM simulations can be executed in parallel with a level-1 granularity and each FVCOM simulation can be executed in parallel with a level-2 granularity. Thus, the proposed COSSD method embraced with the super computer is executed in a hierarchical parallel way. The main task on the master node is blocked until the the FVCOM simulation tasks on slave nodes are all completed. The rest tasks including the evaluation, PDF updating and stop criteria determination are performed on the master nodes.

## Results

Targeting to research on a severe practical accident, Penglai $19-3$ oilfield accident in 2011, Bohai sea in east of China is chosen to conduct experiments in this paper. In this section, the experiment setup information and the experiment details are introduced as follows.

**Figure 6:**
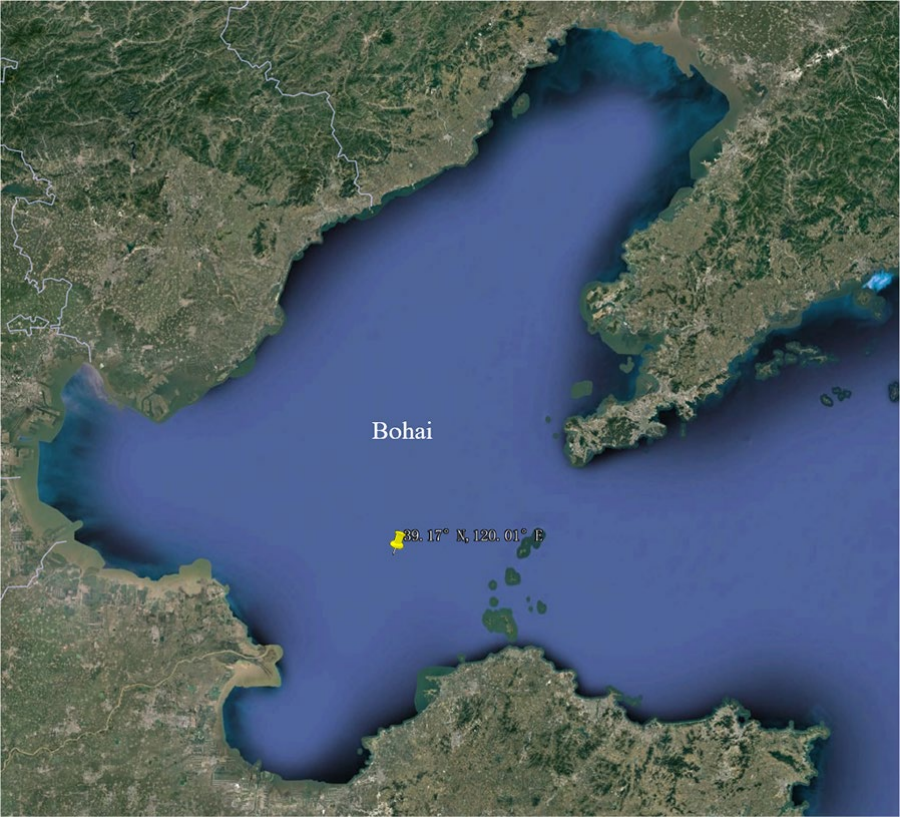
Bohai sea area from google earth. Imagery/Maps data: Google Earth, TerraMetrics and TMap Mobility^26^.

### Experimental setup

To fully describe the experiments in details, the study region, which is Bohai sea and the super computer, which is Tianhe-2 are introduced in following subsections.

#### Region of study

The Penglai $19-3$ oil field is located in the south of central Bohai Sea 11/05 contract area, the Tan-Lu fault zone as shown in Fig. 6. This area is at end of the Bonan uplift^27^. The exploration range of the oil field is from $117.5^{\circ }$E to $122.5^{\circ }$E in terms of longitude and it is from $37^{\circ }$N to $41^{\circ }$N in terms of latitude. The Bohai Sea is semi-closed with an average water depth of 18 m^28^. The 2011 oil leaks were the first large-scale subsea oil spill in China recent years^29^. According to ConocoPhillips China Statistics, the accident results in the release of approximately 700 barrels of oil and 2500 barrels of mineral oil-based drilling mud onto the seabed^30^. Due to the transmission pipeline laying on the seabed generally, in this experiments, we assume that the depth of the oil spill location is constant. The geological model which are utilized to simulate the process of oil spilling is FVCOM^31^. The FVCOM model can solve the time dependent, three dimensional equations for the conservation of mass, momentum, salt, heat and turbulence quantities in an in-compressible hydrostatic fluid.

In this experiment, the simulation accuracy are defined as the difference between the simulated the oil pollution area on the surface of the seawater and the picture taken by the remote sensing satellite. For instance, assuming that the pollution area on the surface is represented by 10, 000 pixels, and 8000 pixels covers the area of real pollution which is taken by the remote sensing satellite, the accuracy of simulation is $80\%$. Remote sensing data utilized in our experiment are obtained on June 11th, 2011 at Bohai Sea Penglai 19-3 Oil field. Advanced Synthetic Aperture Radar (ASAR), produced by Environmental Satellite (Envisat), is utilized to oil leak extraction. With the Wide Swath (WS) mode, ASAR has better coverage range and radiation accuracy in oil spill detection^32^. A large number of researches attempt to accurately detect oil spill in ASAR^32,33^. Thus, in this paper, a single threshold segmentation method, proposed by Yan, et al. in 2015^18^, is utilized to extract oil leak region.

To verify the effectiveness of the proposed COSSD method, a heuristic algorithm, Particle Swarm Optimization (PSO) method is utilized in this paper. The PSO method is a widely-used evolutionary method which has been widely applied in resources allocation, susceptibility analysis and intelligent diagnosis^34–36^. Besides, the Dynamic remote sensing Data-Driven Application System (DDDAS) based oil spill detection approach is also utilized as a comparison method since this technique is also targeting to solve the oil spill problems in Bohai sea^18^. To illustrate the efficiency of the proposed parallel method, a serial implementation of the proposed method is also execute on a single compute node of the super computer.

**Table 1:** The optimization result of the PSO method and the proposed COSSD method.

Iteration	1	2	3	4	5	Oil leak location coordinate
*PSO*	$0\%$	$72.76\%$	$73.58\%$	$73.58\%$	$73.58\%$	$(120.0581^{\circ }\textrm{E}, 38.5079^{\circ }\textrm{N})$
*COSSD*	$0\%$	$64.13\%$	$100\%$	–	–	$(120.0874^{\circ }\textrm{E}, 38.5023^{\circ }\textrm{N})$

#### Super computer Tianhe-2

To improve the real-time performance of the proposed COSSD method, super computer Tianhe-2 is utilized to improve the computational efficiency significantly. Tianhe-2 is super computer developed by National University of Defense Technology in 2003. Super computer Tianhe-2 composed of about 17, 920 compute nodes^37^. The peak computation times of Tianhe-2 can up to $5.49 \times 10^{16}$ times per second, and the stable computation times can up to $3.39 \times 10^{16}$ times per second^38^. These nodes can be divided into big data nodes and Graphic Processing Units (GPU) nodes which are deployed in the different parts of super computer. In this paper, due to the computational characters being highly CPU relied, all experiments are applied to big data nodes. The big data node includes two processors, which is intel Xeon E5-2692 v2 contains 12-core with 2.2 GHZ and 32 GB memory. To significantly enhance the efficiency of the proposed method, 20 FVCOM cases are distributed on 10 big data nodes for parallel execution. For each FVCOM case, 12 cores are allocated to execute.

**Figure 7:**
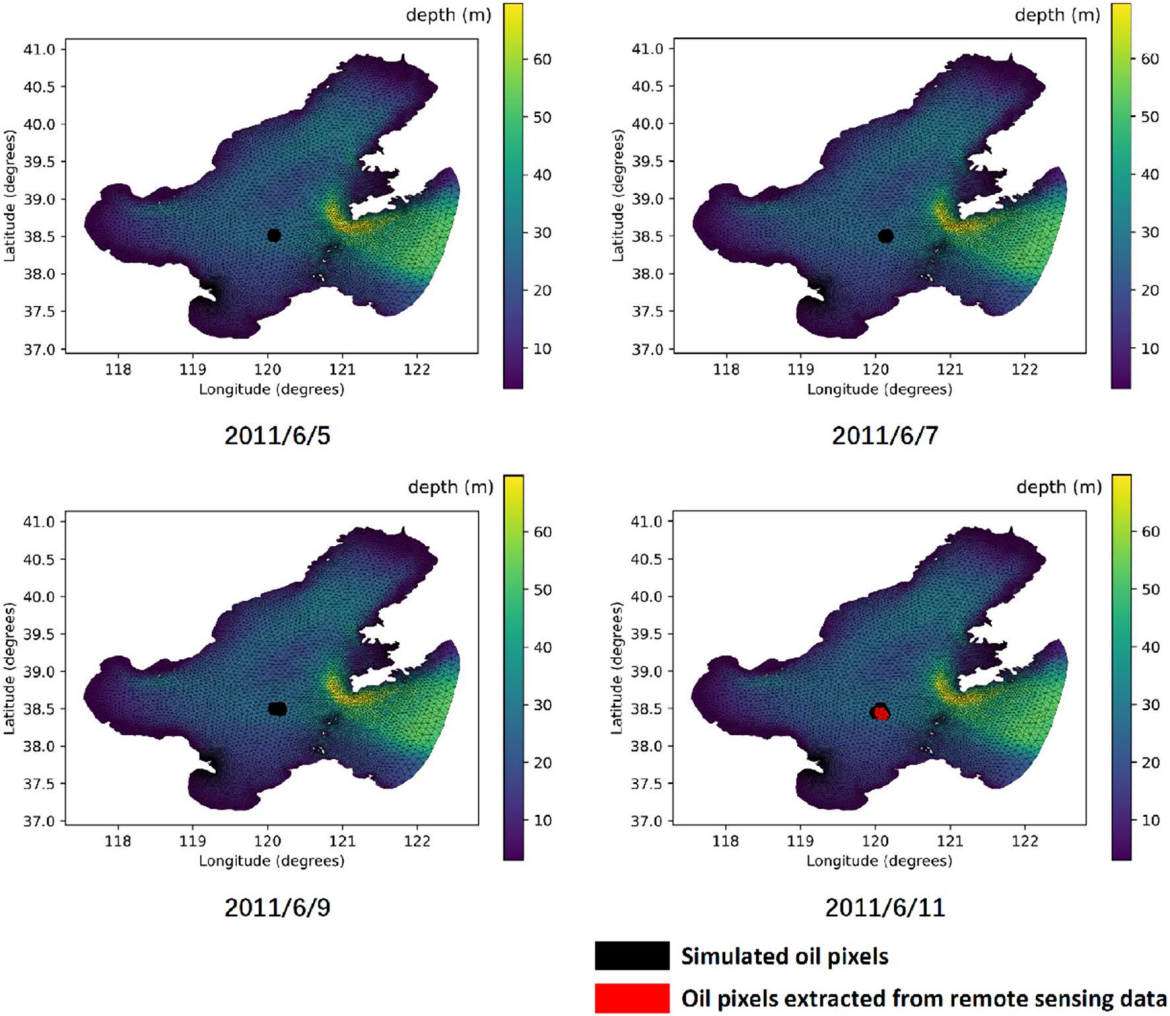
Simulation results of the proposed COSSD method.

### Experiment result

To evaluate the proposed method, the PSO method and DDDAS-based oil spill location detection approach^18^ are utilized to examine the proposed method’s effectiveness and efficiency. We make the following observations.

**Figure 8:**
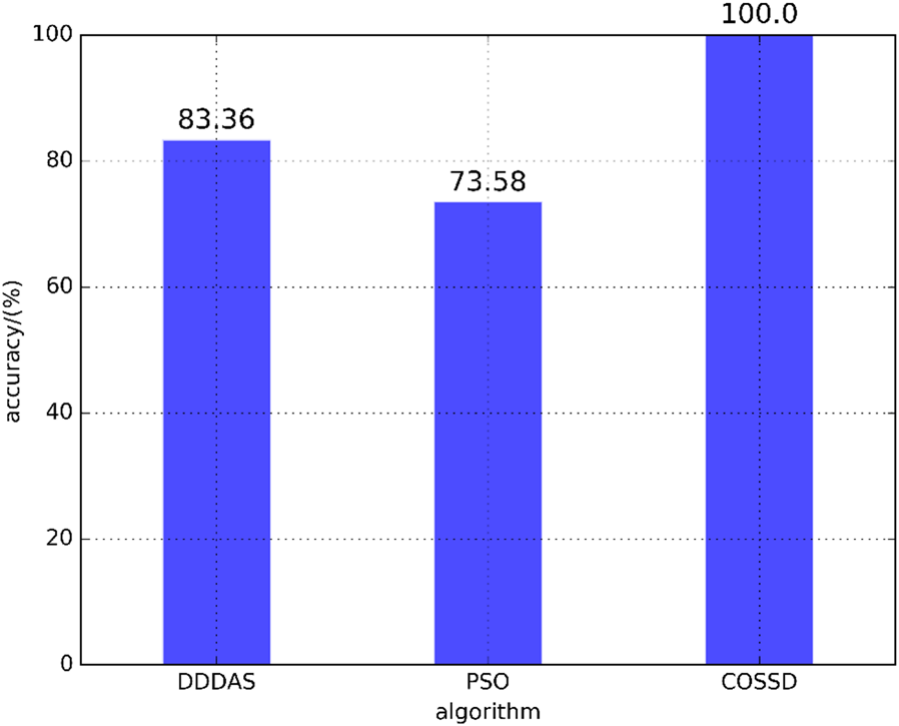
The accuracy comparison of the DDDAS-based oil spill detection approach, the PSO method, and the proposed COSSD method.

**Figure 9:**
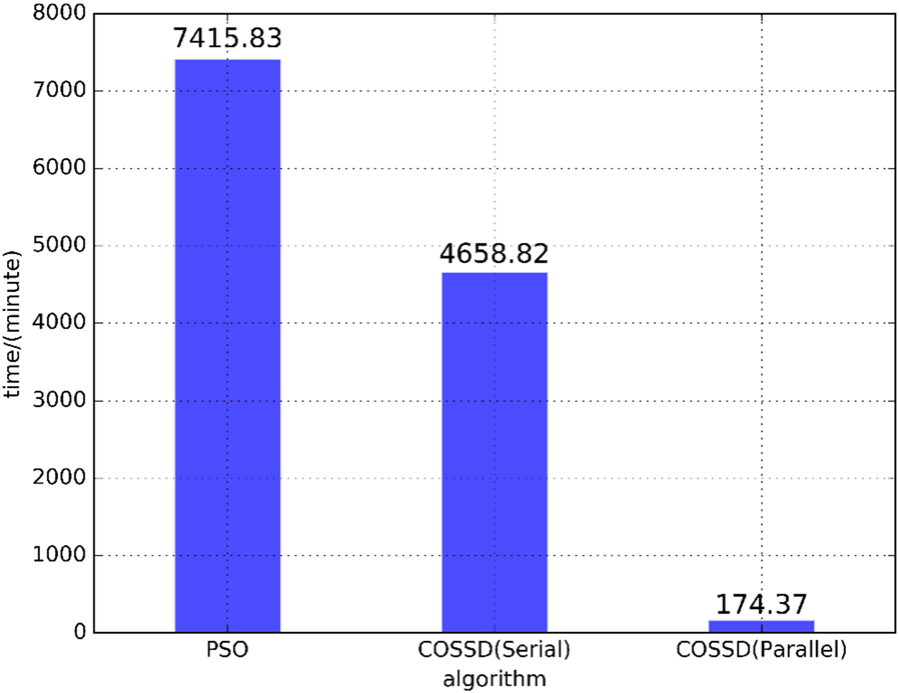
The runtime comparison of the PSO method, a serial implementation of the proposed COSSD method and a hierarchical parallel approach based the proposed COSSD method.

- The effectiveness of the proposed COSSD method has been demonstrated by comparing the simulation results of the proposed algorithm and the remote sensing data. As shown in Fig. 7, from the July 5th, oil particles are iteratively dropped at location $120.0874^{\circ }$E in terms of longitude and $38.5023^{\circ }$N in terms of latitude. The oil film rapidly spread on the surface of the ocean. When the simulated oil spill area is added to the image taken by remote sensing satellite at July 11th, the accuracy of the proposed COSSD method can reach $100\%$.
- The oil spilling location detection accuracy of the DDDAS-based oil leak location detection approach, the PSO method, and the proposed COSSD method is shown in Fig. 8. Note that the accuracy of the DDDAS-based oil spill location detection approach is limited by the prior knowledge of approximate petroleum spilling location while the PSO method and proposed COSSD method search the oil leak source in the whole Bohai sea region. The search range for DDDAS-based oil spill location is from $120^{\circ }01'$E to $120^{\circ }01'$E in longitude and $38^{\circ }17'$N to $38^{\circ }27'$N in latitude. The proposed COSSD method is $\left( 100\% - 83.36\%\right) /83.36\%=19.96\%$ out-performance in accuracy than the DDDAS-based oil spill location detection approach. As shown in Table 1, the PSO method takes 5 iterations to search for a $73.58\%$ accuracy oil leak source location while the proposed COSSD method takes only three iterations and detect a $100\%$ accuracy location. The COSSD is $\left( 100\% - 73.58\%\right) /73.58\% = 35.91\%$ more accurate than the PSO method.
- When comparing the runtime for each algorithm, we guarantee maximum iteration time is 5, and in each iteration, only 20 samples require to process. The stop condition is accomplished 5 iterations, or the accuracy is over $90\%$. Uniform sampling is utilized to generate the particles in the first generation for the PSO method. The proposed COSSD method takes 3 iterations to obtain the $100\%$ accuracy oil leak location, and the PSO method takes 5 iterations only to reach $73.58\%$ accuracy. Note that, $72.76\%$ accuracy is achieved after two generations, but in the next 3 generation, the PSO method can not detect a much better result with over $80\%$ accuracy. The coverage procedure is much more remarkable for the COSSD.
- The oil spilling location detection efficiency of the PSO method, a serial implementation of the proposed COSSD method, and a hierarchical parallel approach based on the proposed COSSD method are verified. The DDDAS-based oil leak location detection approach is not utilized to compare the efficiency because the three-dimensional Estuarine Coastal Ocean Model (ECOM-3d) is utilized to simulate the oil particle moving trajectory, and it can not directly execute on Linux. The ECOM obstacle the experiment on the same platform, which is not comparable. As shown in Fig. 9, the PSO method takes $7415.83/174.37=42.53$ times longer than the hierarchical parallel approach based on the proposed COSSD method. The serial implementation of the proposed COSSD method also takes $4658.82/174.37=26.72$ times longer than the parallel process.

## Conclusions

In this paper, aiming at the emerging service for the offshore petroleum CPS, a COSSD method is developed which can process remote sensing data by exploring the power of rare event probability theory and essential sampling. To gain higher efficiency, the simulation and quality evaluation process are paralleled by a hierarchical parallel approach to deploying the COSSD method on super computer Tianhe-2 to reduce the time consuming of executing the same calculation repeatedly. The remote sensing data which is obtained at the oil spilling accident that happened in Bohai in June 2011 is the test sample to examine the accuracy and efficiency of our research. After 3 times generation, the accuracy is up to $100\%$. With the acceleration of 10 nodes of Tianhe-2, the executing time of simulation and evaluation of 20 generating samples of the hierarchical parallel approach can provide $26.72\times$ speedup than the serial implementation and $42.53\times$ speed up than the PSO method. As the conclusion above, the accuracy and execute time of locating the oil spilling coordinate on remote sensing data utilizing a hierarchical parallel approach for the CE based offshore oil spill detection method on Tianhe-2 for 3 times are $100\%$ and 174.37 minutes.

## Data Availability

All the data used in this study are publicly available. The ASAR for oil leak film extraction is obtained from European Space Agency (https://earth.esa.int/eogateway/instruments/asar). The coastline data and the ocean bathymetry data for numerical simulation are obtained from National Oceanic and Atmospheric Administration (NOAA) National Centers for Environmental Information at https://www.ngdc.noaa.gov/mgg/shorelines/gshhs.html and https://ngdc.noaa.gov/mgg/global/global.html. The output of simulation (in NetCDF format) are available from the corresponding author on reasonable request.

## Code availability

The Python Codes for marine data processing are available on request from Y.W., yuewei.w@cug.edu.cn. The source codes for the oil leak simulation model, FVCOM, are available through personal requests from Marine Ecosystem Dynamics Modeling Lab (http://fvcom.smast.umassd.edu/). The source codes for parallel oil leak source detection are available from authors upon request.

## References

[1] Hao W, Yang T, Yang Q (2021). Hybrid statistical-machine learning for real-time anomaly detection in industrial cyber-physical systems. IEEE Trans. Autom. Sci. Eng..

[2] Zhou J, Li L, Vajdi A, Zhou X, Wu Z (2021). Temperature-constrained reliability optimization of industrial cyber-physical systems using machine learning and feedback control. IEEE Trans. Autom. Sci. Eng..

[3] Chen X (2017). Design automation for interwell connectivity estimation in petroleum cyber-physical systems. IEEE Trans. Comput.-Aided Des. Integr. Circuits Syst..

[4] Chen, X. *et al.* Analysis of production data manipulation attacks in petroleum cyber-physical systems. in *Proceedings of the 35th International Conference on Computer-Aided Design*. 1–7 (2016).

[5] Wang F, Yuan H (2010). Challenges of the sensor web for disaster management. Int. J. Digit. Earth.

[6] Rink K (2018). Virtual geographic environments for water pollution control. Int. J. Digit. Earth.

[7] Zhang Z (2019). A cybergis-enabled multi-criteria spatial decision support system: A case study on flood emergency management. Int. J. Digit. Earth.

[8] Elhakeem A, Elshorbagy RCW (2007). Oil spill simulation and validation in the Arabian (Persian) gulf with special reference to the UAE coast. Water Air Soil Pollut..

[9] Mu L, Zhao E, Wang Y, Zomaya A (2020). Buoy sensor cyberattack detection in offshore petroleum cyber-physical systems. IEEE Trans. Serv. Comput..

[10] Zhuge H, Xing Y (2011). Probabilistic resource space model for managing resources in cyber-physical society. IEEE Trans. Serv. Comput..

[11] Rajendran S (2021). Monitoring oil spill in Norilsk, Russia using satellite data. Sci. Rep..

[12] Chaudhary V, Kumar S (2021). Dark spot detection for characterization of marine surface slicks using UAVSAR quad-pol data. Sci. Rep..

[13] García-Sánchez G, Mancho AM, Ramos AG, Coca J, Wiggins S (2022). Structured pathways in the turbulence organizing recent oil spill events in the eastern Mediterranean. Sci. Rep..

[14] Liu Y, Weisberg R, Hu C, Zheng L (2011). Tracking the deepwater horizon oil spill: A modeling perspective. Eos Trans. Am. Geophys. Union.

[15] Zodiatis G, Lardner R, Solovyov D, Panayidou X, Dominicis MD (2012). Predictions for oil slicks detected from satellite images using MyOcean forecasting data. Ocean Sci..

[16] De Dominicis M, Pinardi N, Zodiatis G, Lardner R (2013). Medslik-II, a Lagrangian marine surface oil spill model for short-term forecasting—part 1: Theory. Geosci. Model Dev..

[17] Dominicis MD, Archetti NPGZ (2013). Medslik-II, a Lagrangian marine surface oil spill model for short-term forecasting—Part 2: Numerical simulations and validations. Geoscience.

[18] Yan J, Wang L, Chen L, Zhao L, Huang B (2015). A dynamic remote sensing data-driven approach for oil spill simulation in the sea. Remote Sens..

[19] Chen, X. *et al.* Offshore oil spill monitoring and detection: Improving risk management for offshore petroleum cyber-physical systems: (invited paper). in *2017 IEEE/ACM International Conference on Computer-Aided Design, ICCAD 2017, Irvine, CA, USA, November 13–16, 2017* (Parameswaran, S. ed.). 841–846 (IEEE, 2017).

[20] Chen C, Liu H, Beardsley RC (2003). An unstructured grid, finite-volume, three-dimensional, primitive equations ocean model: Application to coastal ocean and estuaries. J. Atmos. Ocean. Technol..

[21] Chen C (2006). Fvcom User Manual.

[22] Zhao G, Niu X (2022). Local amplification of tsunami waves along the west coast of Negros Island and Panay Island. Appl. Ocean Res..

[23] Rubinstein R (1999). The cross-entropy method for combinatorial and continuous optimization. Methodol. Comput. Appl. Probab..

[24] Graça, D. S. & Zhong, N. Computability of differential equations. in *Handbook of Computability and Complexity in Analysis*. 71–99 (Springer, 2021).

[25] Selivanova, S. Computational complexity of classical solutions of partial differential equations. in *Revolutions and Revelations in Computability: 18th Conference on Computability in Europe, CiE 2022, Swansea, UK, July 11–15, 2022, Proceedings*. 299–312 (Springer, 2022).

[26] Maps, G. *Google Maps*. https://www.google.com/maps/@37.1328919,119.8673427,7z?hl=en. Accessed 4 Mar 2022 (2022).

[27] Liu, X. *et al.* Modelling of oil spill trajectory for 2011 Penglai 19-3 coastal drilling field, China. *Appl. Math. Model.*10.1016/j.apm.2014.10.063 (2014).

[28] Lu Y, Wang QTX, Zheng G, Li X (2013). Determining oil slick thickness using hyperspectral remote sensing in the Bohai Sea of China. Int. J. Digit. Earth.

[29] Ivshina IB, Kuyukina MS, Krivoruchko AV (2015). Oil spill problems and sustainable response strategies through new technologies. Environ. Sci. Process. Impacts.

[30] State, Oceanic & Administration. Bulletin of China’s marine environmental status of China for the year of 2010. *Contemp. Chin. Popul. English Version* **30**, 16 (2011).

[31] Cowles GW (2008). Parallelization of the FVCOM coastal ocean model. Int. J. High Perform. Comput. Appl..

[32] Alpers W, Holt B, Zeng K (2017). Oil spill detection by imaging radars: Challenges and pitfalls. Remote Sens. Environ..

[33] Brekke C, Solberg AH (2008). Classifiers and confidence estimation for oil spill detection in Envisat ASAR images. IEEE Geosci. Remote Sens. Lett..

[34] Deng, W., Xu, J., Zhao, H. & Song, Y. A novel gate resource allocation method using improved PSO-based QEA. *IEEE Trans. Intell. Transport. Syst.* (2020).

[35] Moayedi H, Mehrabi M, Mosallanezhad M, Rashid ASA, Pradhan B (2019). Modification of landslide susceptibility mapping using optimized PSO-ANN technique. Eng. Comput..

[36] Deng W, Yao R, Zhao H, Yang X, Li G (2019). A novel intelligent diagnosis method using optimal LS-SVM with improved PSO algorithm. Soft Comput..

[37] Peng, Z., Lu, Y., Cheng, Z. & Du, Y. A low communication overhead breadth-first search based on global bitmap. in *International Conference on Algorithms and Architectures for Parallel Processing*. 114–129 (Springer, 2018).

[38] Wang, C., Cai, T., Suo, G., Lu, Y. & Zhou, E. Distforest: A parallel random forest training framework based on supercomputer. in *2018 IEEE 20th International Conference on High Performance Computing and Communications; IEEE 16th International Conference on Smart City; IEEE 4th International Conference on Data Science and Systems (HPCC/SmartCity/DSS)*. 196–204 (IEEE, 2018).

